# The relationship between entomological indicators of *Aedes aegypti* abundance and dengue virus infection

**DOI:** 10.1371/journal.pntd.0005429

**Published:** 2017-03-23

**Authors:** Elizabeth A. Cromwell, Steven T. Stoddard, Christopher M. Barker, Annelies Van Rie, William B. Messer, Steven R. Meshnick, Amy C. Morrison, Thomas W. Scott

**Affiliations:** 1 Department of Epidemiology, University of North Carolina, Gillings School of Global Public Health, Chapel Hill, North Carolina, United States of America; 2 Graduate School of Public Health, San Diego State University, San Diego, California, United States of America; 3 Department of Entomology and Nematology, University of California, Davis, Davis, California, United States of America; 4 Division of Infectious Diseases, Oregon Health and Science University, Portland, Oregon, United States of America; Duke-NUS GMS, SINGAPORE

## Abstract

Routine entomological monitoring data are used to quantify the abundance of *Ae*. *aegypti*. The public health utility of these indicators is based on the assumption that greater mosquito abundance increases the risk of human DENV transmission, and therefore reducing exposure to the vector decreases incidence of infection. Entomological survey data from two longitudinal cohort studies in Iquitos, Peru, linked with 8,153 paired serological samples taken approximately six months apart were analyzed. Indicators of *Ae*. *aegypti* density were calculated from cross-sectional and longitudinal entomological data collected over a 12-month period for larval, pupal and adult *Ae*. *aegypti*. Log binomial models were used to estimate risk ratios (RR) to measure the association between *Ae*. *aegypti* abundance and the six-month risk of DENV seroconversion. RRs estimated using cross-sectional entomological data were compared to RRs estimated using longitudinal data. Higher cross-sectional *Ae*. *aegypti* densities were not associated with an increased risk of DENV seroconversion. Use of longitudinal entomological data resulted in RRs ranging from 1.01 (95% CI: 1.01, 1.02) to 1.30 (95% CI: 1.17, 1.46) for adult stage density estimates and RRs ranging from 1.21 (95% CI: 1.07, 1.37) to 1.75 (95% CI: 1.23, 2.5) for categorical immature indices. *Ae*. *aegypti* densities calculated from longitudinal entomological data were associated with DENV seroconversion, whereas those measured cross-sectionally were not. *Ae*. *aegypti* indicators calculated from cross-sectional surveillance, as is common practice, have limited public health utility in detecting areas or populations at high risk of DENV infection.

## Author summary

In this study, we compared measures of entomological risk collected through routine household entomological monitoring by estimating an association with human DENV infection. Longitudinal entomological and human serology data from Iquitos, Peru, were used to test associations between *Ae*. *aegypti* indices and the 6-month risk of DENV seroconversion. Our analysis found no association between cross-sectional measures of *Ae*. *aegypti* abundance and the risk of DENV seroconversion. Longitudinal measures of *Ae*. *aegypti* were better proxies for DENV risk, primarily among adult stage mosquito indicators. DENV transmission is complex and time-varying; the relationship between vector density and risk is not static nor adequately characterized through periodic entomological surveillance. While entomological monitoring will continue to serve a role in the evaluation of vector control interventions (e.g., comparing pre- and post-intervention abundance), our analysis challenges the validity of most *Ae*. *aegypti* indicators as adequate proxies for true DENV exposure risk.

## Introduction

Dengue virus (DENV), which is transmitted by the bite of female *Aedes aegypti* mosquitoes, causes more human morbidity and mortality than any other arthropod-borne virus [1]. Since the 1950s, dengue has spread via the globalization of trade and travel, rapid urbanization and expansion of vector habitats [2]. The four serotypes (DENV1, DENV2, DENV3 and DENV4) occur throughout the tropics and infect approximately 390 million persons per year [1]. Until effective DENV vaccines become broadly commercially available, vector control will remain the primary prevention strategy in most dengue endemic settings [3] and even as vaccines become accessible vector control will be needed to supplement vaccine efforts [4], as well as control of other arboviruses also vectored by *Ae*. *aegypti*.

The World Health Organization recommends monitoring vector abundance for the targeting and evaluation of vector control interventions [5]. *Ae*. *aegypti* monitoring was first employed in yellow fever control programs in the first half of the 20^th^ century [6, 7]. Since then, over two dozen indicators have been proposed to quantify abundance of *Ae*. *aegypti*. Entomological monitoring data are typically collected from households sampled from neighborhoods or blocks on a routine or ad hoc basis [8]. The frequency of entomological data collection also varies by setting, and WHO guidelines recommend implementation occur at a frequency from “weeks to months” [5]. As such, entomological monitoring surveys impose cross-sectional measurement of the highly dynamic *Ae*. *aegypti* population. Monitoring indicators vary by mosquito life stage (adults, larvae and/or pupae), availability of larval development sites (infestation indices), and process of collection (fixed trap or human-based surveys such as adult aspirator collections, household inspection for larvae) [9]. The public health utility of these indicators is based on the assumption that greater mosquito abundance increases the risk of DENV transmission, and therefore reducing exposure to the vector decreases incidence of infection. Further, by identifying “hot spots” of *Ae*. *aegypti* infestation, targeted vector control would be an efficient use of limited intervention resources [10].

To date, studies have not shown a consistent association between various indices and infection or disease outcomes [7]. This may be due to several limitations inherent to the large-scale measurement of *Ae*. *aegypti* densities. First, there is no established threshold of *Ae*. *aegypti* density associated with an increased risk of human DENV infection [11]. Second, entomological survey techniques may not capture the fine spatial and temporal variability in an urban setting due to the constraints dictated by household-based monitoring, and the fact that indices are calculated from cross-sectional prevalence measures, not derived from continuous monitoring. Third, while adequate sampling of immature and adult populations requires consideration of vector dynamics [12] and spatial relationships [13], the data do not capture the daily productivity of individual larval development sites or the activity of individual mosquitoes over their lifespan [8, 14]. Finally, previous attempts to quantify the association between vector abundance and dengue outcomes may also have been biased due to measurement error caused by operational constraints and collection procedures [9], and methodological issues, such as restricting the analysis outcomes to infected people who sought treatment or small sample size [7].

*Ae*. *aegypti* densities may also fail to describe risk of DENV infection due to the complexity of transmission. The probability of transmission is dependent on human movement to introduce DENV into mosquito populations and the presence of susceptible individuals that mosquitoes infect to perpetuate new rounds of transmission [15]. Because *Ae*. *aegypti* are daytime-biting mosquitoes that are highly adapted to the human urban environment [16], their frequent biting contact with susceptible human hosts is mediated by social and economic [17] factors that govern human movement through times and spaces where they encounter mosquitoes [18]. While high concentrations of *Ae*. *aegypti* within or around a household present an opportunity for clustered DENV transmission, it ignores transmission occurring in other places [19, 20].

To help predict risk and direct public health interventions, there is substantial interest in an improved understanding of the utility of *Ae*. *aegypti* monitoring measures in terms of an association with DENV infection, according to mosquito life stage and spatial scale of measurement. We aimed to systematically examine measures of entomological risk collected through routine household surveillance with human DENV infection using longitudinal entomological and human serology data to test associations between *Ae*. *aegypti* indices and the 6-month risk of DENV seroconversion.

## Methods

### Ethics statement

Written informed consent (and assent for children 8–17 years of age) was obtained for all individuals providing serological data. Written informed consent was provided by parents or guardians for children under 18 years of age. Written consent (1999–2003) or oral consent (2008–2010) was obtained from an adult head of household for entomological surveys as approved by the institutional review boards. Oral consent for entomological surveys was documented upon obtaining access to the household and heads of households were provided information sheets describing the data collection procedures. Data collection procedures were approved by the University of California, Davis (Protocols 2002–10788 and 2007–15244), Instituto Nacional de Salud, and Naval Medical Research Center Institutional Review Boards (Protocols NMRCD.2001.0008 and NMRCD2007.0007). This ancillary analysis was approved by the Institutional Review Board at the University of North Carolina at Chapel Hill (Study # 14–3151).

### Study site

The analytical cohort was constructed using entomological and serological data collected between 1999–2003 and 2008–2010 from two longitudinal cohort studies implemented in Iquitos, Peru. Iquitos, the largest city in the Peruvian Amazon, has a population of approximately 350,000 [21]. DENV1 is presumed to have been introduced in 1990–1991 [22], followed by DENV2 in 1995 [23], DENV3 in 2001 [24], and DENV4 in 2008 [25]. Seasonal epidemic levels of DENV transmission occurred throughout this period [21, 25]. From 1999–2003, study activities were implemented in four city districts: Maynas, Punchana, Belen and San Juan. During the period 2008–2010, data were collected from two neighborhoods: Maynas and Tupac Amaru (located within the Maynas and Punchana districts).

### Entomological and sociodemographic data collection

Procedures for entomological data collection were previously described [13, 21]. Briefly, once households were enrolled, two-person study teams collected entomological data following a circuit to survey neighboring households on the same and/or neighboring block (with block defined as a group of households that shared a common perimeter defined by city streets) within an approximately two-week period. The entire study area required approximately four months to complete data collection, upon which entomological surveys resumed following the same schedule.

Adult *Ae*. *aegypti* were collected using CDC backpack aspirators (1999–2009) [26] or Prokopack aspirators (2009–2010) [27] in both the exterior and interior of the participating household by passing the vacuum tube over common *Ae*. *aegypti* resting sites, outside walls, vegetation, and the entrance of potential larval habitats. Pupae and larvae were collected via enumeration of all wet containers or other larval development sites that contained water upon inspection. During surveys, all observed pupae and a sample of larvae were collected in small plastic Whirlpack bags; larval density was estimated as one of four levels (0, 1–10, 11–100, >100). All adult, larval and pupal samples were transported to and examined at the study laboratory, counted and identified to species and sex. Pupal data were recorded as observed counts. The total number of adult male and female *Ae*. *aegypti* mosquitoes collected in the interior and exterior of the dwelling were recorded. Household demographic data were collected for variables including enumeration of household residents by age and sex, household water source, sanitation facility, presence of electricity, type of building material, roof structure, and any reported use of insecticide or larvacide.

### Indicators for *Ae*. *aegypti* density

The indicators were classified by scale (household or block) and life stage (adult, pupal and/or larval). Household-level indicators were calculated using the observed survey data. To construct block-level indicators, all household survey data were first aggregated by block using a unique block identification number and circuit schedule. Indicators were then calculated using the aggregated block-level *Ae*. *aegypti* data. Block-level measures were then linked back to individual households by matching on block identifier and date of collection. The household-level indicators and their definitions are summarized in Table 1 and block-level indicators are summarized in Table 2.

**Table 1:** Summary of household-level indicators of *Aedes aegypti* tested for an association with seroconversion to DENV

Indicator^a^	Definition/Formula	*Aedes aegypti* Life stage	Variable type
Adult *Ae*. *aegypti* in the household	Number observed	Adult	Continuous
	Exposed defined as any adults observed (>0)	Adult	Categorical
Adult female *Ae*. *aegypti* in the household	Number observed	Adult	Continuous
	Exposed defined as any adult females observed (>0)	Adult	Categorical
Presence of adult *Ae*. *aegypti* indoors[28]	Number observed	Adult	Continuous
	Exposed defined as any adult indoors (>0)	Adult	Categorical
Presence of adult female *Ae*. *aegypti*[28] indoors	Number observed	Adult	Continuous
	Exposed defined as any adult female indoors (>0)	Adult	Categorical
Single Larval Method[29]	# containers with ≥1 larvae	Larvae	Continuous
	Exposed defined as SLM >0	Larvae	Categorical
Presence of pupae in the household	Exposed defined as any pupae observed in containers (>0)	Pupae	Categorical
Pupae per Hectare[14]	# pupae/household area measured in hectare	Pupae	Continuous
Pupae per Person[14]	# pupae/household population	Pupae	Continuous
Container Index (Receptacle Index)[6]	# of containers infested with larvae or pupae/ total number of containers inspected x 100%	Pupae, Larvae	Continuous
	Exposed defined as CI >0	Pupae, Larvae	Categorical
*Stegomyia* Index[30]	# positive containers (larvae or pupae)/population x 1000	Pupae, Larvae	Continuous
	Exposed defined as SI >0	Pupae, Larvae	Categorical

^a^Parentheses signify different names for the same indicator.

**Table 2:** Summary of block-level indicators of *Aedes aegypti* tested for an association with seroconversion to DENV

Indicator^a^	Definition/Formula	*Aedes aegypti* Life stage	Variable type
Breteau Index[5]	(# containers infested/total households) x 100	Pupae, Larvae	Continuous
	Exposed defined as BI ≥ 5	Pupae, Larvae	Categorical
House Index (Premise Index; *Aedes* Index) [6]	(# households infested /total households) x 100%	Pupae, Larvae	Continuous
	Exposed defined as HI ≥ 5	Pupae, Larvae	Categorical
Adult Premise Index[9]	# premises positive for adult females/#premises x 100	Adult	Continuous
	Exposed defined as APrI ≥ 5	Adult	Categorical
Adult Density Index[9]	# adult females / # of premises	Adult	Continuous
	Exposed defined as ADI > 0	Adult	Categorical
Pupa Index[28]	(# pupae/total number households inspected) x 100	Pupae	Continuous
	Exposed defined as PI > 5	Pupae	Categorical
Pupae per Hectare[14]	# pupae/household area (hectare)	Pupae	Continuous
Pupae per Person[14]	# pupae/household population	Pupae	Continuous
Infested Receptacle Index[31]		# positive containers/total number of households	Pupae, Larvae	Continuous
	Exposed defined as IRI > 0	Pupae, Larvae	Categorical
Container Index (Receptacle Index)[6]	# containers infested/total number of containers inspected x 100%	Pupae, Larvae	Continuous
	Exposed defined as CI ≥ 5	Pupae, Larvae	Categorical
Potential Container Index[32]	(# potential breeding sites + # positive breeding sites)/households inspected	Pupae, Larvae	Continuous
	Exposed defined as PCI≥ 2	Pupae, Larvae	Categorical
*Stegomyia* Index[30]	# positive containers (larvae or pupae)/population x 1000	Pupae, Larvae	Continuous
	Exposed defined as SI > 0	Pupae, Larvae	Categorical

^a^Parentheses signify different names for the same indicator.

Since the distribution of *Ae*. *aegypti* counts across all life stages is narrow in most settings, we dichotomized the continuous indicators to determine if categorical characterization of mosquito abundance would reflect a better fit to the data. To test categorical (dichotomous) versions of continuous indicators, a preliminary analysis was conducted to identify cut-off values by estimating the sensitivity and specificity of the mosquito density in terms of DENV infection at different levels (data not presented). There is no consensus in the literature as to what categorical values of mosquito density measures correlate with DENV infection, therefore we used the following systematic approach to select categories and then test for an association. This approach was used to allow the distribution of the continuous indicator value to inform categorization without data mining for an association. To choose a categorical cutpoint, the sensitivity of the mosquito density indicator to identify a DENV seroconversion was calculated for increments of five (e.g., a Breteau Index of 0, 5, 10, etc.), with the exception of the Potential Container Index, which was estimated for increments of two. The cutpoint was chosen as >0 if the sensitivity was less than 50% at that value. If cutpoints greater than zero had a sensitivity >50%, then the cutpoint (not zero) with the highest sensitivity was chosen for evaluation. Once a categorical variable was defined, it was then tested for an association with risk of DENV seroconversion. Categorical classification of continuous indicators tested is listed in Tables 1 and 2. Data on eggs or exact larval counts were not collected in the parent study; therefore, indices relying on this information could not be tested.

Cross-sectional entomological indicators were calculated using vector data from a single entomological survey observation. Longitudinal household-level indicators were calculated as an average of entomological data observed within the 12 months preceding the start of the seroconversion interval (up to three survey visits collected approximately every four months). If a paired sample interval began before any entomological data collection, the cross-sectional measure of mosquito density was used. For block measures, indicators were calculated by averaging block-level densities calculated from surveys conducted within 12 months from the start of the seroconversion interval.

### Serological data collection and outcome classification

In the parent study, members of households selected for entomological monitoring were asked to provide blood samples every six months [21, 33]. Samples were collected at the participant’s home, stored in ice and transported to the study laboratory within four hours of collection. Sera were tested at two (1999–2003) and four (2008–2010) serum dilutions plaque reduction neutralization test (PRNT) [34] at the United States Naval Medical Research Unit No. 6 laboratory in Lima, Peru. To identify seroconversion to DENV, a serum sample was considered positive for DENV if a dilution neutralized 70% of the test virus (PRNT_70_) [21, 33].

The primary outcome of interest in this analysis was seroconversion to any circulating DENV serotypes as determined by PRNT_70_. The longitudinal serological samples used in this analysis were previously reviewed to determine seroconversion [33]. In brief, to minimize misclassification of serological data, the full serological profile of subjects was reviewed as follows: if the increase in titer that reduced DENV plaques between a negative sample and a subsequent sample was at least 20% and all subsequent samples were positive, the subject was determined to have seroconverted. However, if subsequent PRNT results were not consistent with respect to seroconversion (e.g., negative-positive-negative), the subject was classified as not having seroconverted. For this study, serological results for all paired samples were classified as a binary outcome (any seroconversion versus no seroconversion).

### Construction of analytical cohort

**Fig 1:**
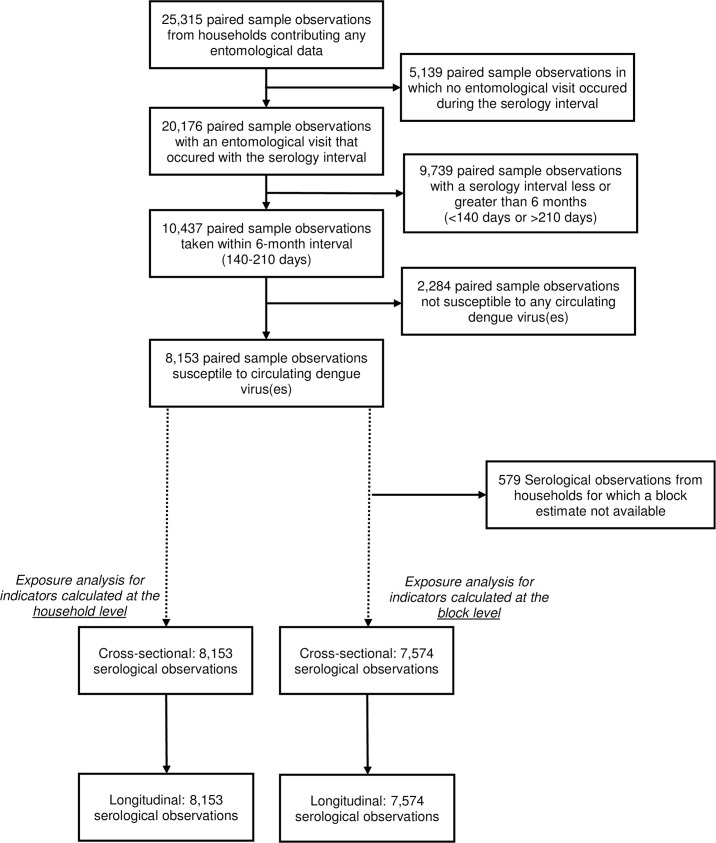
Construction of the Analysis Dataset. Fig 1 presents the construction of the analytical cohort based on inclusion criteria.

Fig 1 illustrates the construction of the analysis cohort. Serological data were reviewed to identify paired sample observations taken approximately six months apart that could be linked to household entomological data. To account for operational constraints around serology collection, the at-risk interval was defined as 140 to 220 days. Each paired sample interval for which a subject was susceptible to any of the circulating DENV serotypes (DENV1 and DENV2: all study years; DENV3: 2001–2010; DENV4, 2008–2010) was included in the risk set.

**Fig 2:**
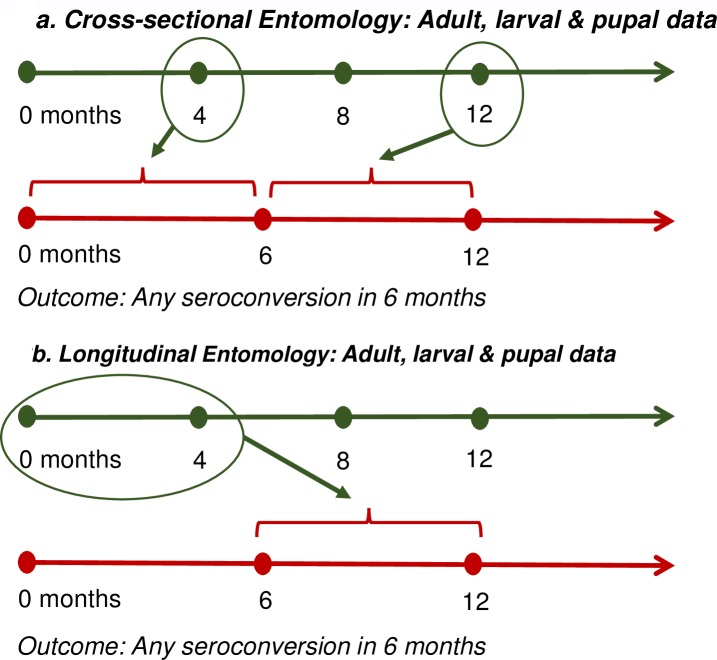
Calculation of cross-sectional and longitudinal measures of vector abundance. Fig 2a (top) illustrates how cross-sectional measures of vector density were assigned to a six-month paired serological sample. In Figure 2b (bottom), longitudinal measures of vector density were calculated by taking an average of indices calculated from survey observations in the period up to 12 months preceding the seroconversion interval. **Captions for panel in Figure 2a.** The entomological measurement taken within the seroconversion interval (and nearest the end of the interval, if there was more than one), was used in the regression analysis as the entomological indicator variable. Other approaches to linking a single entomological survey observation with serological data are presented in the SI. The risk ratios estimate the effect of the mosquito indicator on the 6-month risk of DENV infection. Since inapparent was measured using paired sample serology, this outcome is inherently interval censored. **Caption for panel in Figure 2b.** Entomological indices collected up to 12 months before the start of the seroconversion interval were averaged and used in the regression analysis as the entomological indicator variable, matched to a single 6-month paired sample interval.

For household-level indicators, entomological data were matched by the date nearest the end of (but within) each paired serological sample interval. For block-level indicators, datasets were constructed by restricting to serological observations from blocks in which at least five households were surveyed, using the month and year of block data collection to anchor in time block-level densities to serology. Finally, longitudinal densities were calculated by averaging entomological data collected in the 12 months preceding the start of the seroconversion interval. Fig 2 illustrates how cross-sectional and longitudinal measures of vector abundance were calculated and linked to the 6-month seroconversion paired sample interval.

### Statistical analysis

The association between each *Ae*. *aegypti* indicator and the 6-month risk of DENV seroconversion was estimated using a log binomial generalized estimating equation (GEE) [35], separately for each household-level and block-level indicator, and for both the cross-sectional and longitudinal scenarios. The log link with a binomial distribution allowed for estimation of risk ratio point estimates by exponentiating the beta coefficient for the indicator variable and calculation of 95% confidence intervals (CI) [36]. For models using household-level densities, the GEE accounted for clustering due to repeated individual measures and dependence due to household membership using an exchangeable correlation structure; models for block-level densities accounted for repeated observations from individuals and block level membership. *A priori*, we chose dengue transmission season, participant age and sex as confounding variables for use in all adjusted analyses of household-level indicators and season, participant age (dichotomized at 18 years), and any reported use of larvacide by the head of household for adjustment of all block-level indicators. Dengue transmission season was defined as by the start of the seroconversion interval (May-August (reference group), September-December, January-April). All analyses were conducted in SAS/STAT software, version 9.4 of the SAS system for Windows (SAS Institute, Cary, NC).

### Sensitivity analyses

Sensitivity analyses were conducted to account for possible bias resulting from construction of the dataset. The objective of the sensitivity analysis was to determine if the adjusted risk ratios were sensitive to decisions made to construct the analytical dataset. To implement these analyses, the same method as described in the main analysis was employed. First, different inclusion criteria for serological observations was used to test more restrictive or relaxed scenarios, as well as stratification by study years. Second, sensitivity analyses included alternate strategies for linking serology to entomology. Third, vector densities were calculated from entomological data 6 months prior to serology compared to 12 months prior to serology. Finally, the analysis was stratified by aspirator type used during data collection.

### Ethical considerations

Written informed consent (and assent for children 8–17 years of age) was obtained for all individuals providing serological data. Written informed consent was provided by parents or guardians for children under 18 years of age. Written consent (1999–2003) or oral consent (2008–2010) was obtained from an adult head of household for entomological surveys as approved by the institutional review boards. Oral consent for entomological surveys was documented upon obtaining access to the household and heads of households were provided information sheets describing the data collection procedures. Data collection procedures were approved by the University of California, Davis (Protocols 2002–10788 and 2007–15244), Instituto Nacional de Salud, and Naval Medical Research Center Institutional Review Boards (Protocols NMRCD.2001.0008 and NMRCD2007.0007). This ancillary analysis was approved by the Institutional Review Board at the University of North Carolina at Chapel Hill (Study # 14–3151).

## Results

In total, 13,526 households contributed 90,330 entomological observations and 25,755 paired serological samples (from 6,775 individuals). A total of 20,176 serological observations could be linked to entomological data. Fig 1 details the analytical sample size. For the cross-sectional household-level analysis, 4,089 household entomological observations (from 1,377 unique households) were linked to 8,153 paired blood samples (from 3,824 individuals). For the longitudinal household-level analysis, 15,548 entomological observations from those 1,377 households were used to calculate average densities and matched to the 8,153 serological observations. The same set of serological and entomological observations were included in the block-level analyses, with the exception of 579 serological observations for which a block density could not be obtained (<5 households per block-visit were surveyed). A total of 7,574 serological paired samples (from 3,644 individuals) were used in the cross-sectional and longitudinal block-level analyses.

The mean age of individuals at first paired sample was 20.9 years (standard deviation: 16.3, range 2–96) and 57.7% of subjects were female. At first study visit, most households contributing any serological data reported access to electricity (99.7%), piped sanitation (77.1%), and potable water (75.2%), had open or partially open household roof structure (93.0%), and were constructed from either mud and/or wood (49.2%) or concrete and/or brick (50.8%). Only 28.2% of households reported using Abate (larvacide) at enrollment. There were a total of 1,191 seroconversions (14.6%) in the analysis of household level indicators and 1,129 seroconversions (14.9%) in the analysis of block-level indicators. Tables 3 and 4 present the distribution of entomological indicators.

**Table 3:** Distribution of continuous entomological monitoring indicators for serological observations

	Cross-sectional							Longitudinal (12 months)						
Indicator	N	Mean	SD	Min	Q1	Q3	Max	N	Mean	SD	Min	Q1	Q3	Max
**Continuous Measures**		** **	** **	** **										
*Household level* ^a^														
Adult *Ae*. *aegypti*	8153	0.7	3.6	0	0	0	163.0	8153	0.5	1.8	0	0	0.5	84.0
Adult female *Ae*. *aegypti*	8153	0.4	1.7	0	0	0	51.0	8153	0.3	1.04	0	0	0.3	47.0
Single Larval Method	8153	0.2	0.6	0	0	0	9.0	8145	0.2	0.5	0	0	0.3	6.8
Pupae in household containers	8153	1.7	15.9	0	0	0	642.0	8148	2.1	11.1	0	0	0	289.0
Pupae per Hectare	8153	114.6	1154.4	0	0	0	62879.5	8153	180.6	1639.9	0	0	0	62276.3
Pupae per Person	8139	0.24	2.5	0	0	0	78.0	8143	0.3	3.0	0	0	0	108.7
Container Index	8153	4.3	13.0	0	0	0	100.0	8153	0.4	3.9	0	0	0.1	100.0
*Stegomyia* Index	8133	0.03	0.1	0	0	0	2.0	8133	0.03	0.1	0	0	0.04	1.5
*Block level* ^b^														
Breteau Index	7574	22.1	23.3	0	5.1	32.7	214.3	7574	26.1	22.7	0	9.4	36.8	404.5
House Index	7574	14.4	12.4	0	4.5	22.0	63.6	7574	16.3	10.7	0	7.5	23.6	63.0
Adult Premise Index	7574	15.3	12.4	0	5.9	23.1	88.9	7574	14.0	8.3	0	7.5	19.4	50.0
Adult Density Index	7574	0.3	0.4	0	0.1	0.4	8.8	7574	0.3	0.3	0	0.1	0.3	3.8
Pupa Index	7574	186.0	418.4	0	0	197.1	9312.5	7574	227.9	345.1	0	39.3	273.3	5283.3
Pupae per Hectare	7574	123.2	279.9	0	0	125.9	7669.7	7574	154.7	242.1	0	26.3	186.8	2611.3
Pupae per Person	7574	0.3	0.6	0	0	0.3	11.9	7574	0.4	0.5	0	0.1	0.4	8.1
Infested Receptacle Index	7574	0.2	0.2	0	0.1	0.3	2.1	7574	0.3	0.2	0	0.09	0.4	4.0
Container Index	7574	5.5	4.9	0	1.7	8.2	33.3	7574	6.2	4.0	0	3.2	8.8	34.5
Potential Container Index	7574	2.8	1.4	0.2	1.7	3.5	9.2	7574	5.9	4.0	0	2.9	8.4	34.5
*Stegomyia* Index	7574	34.1	36.2	0	7.9	49.0	389.6	7574	40.3	35.8	0	15.4	56.7	679.4

^a^ Sample size of serological observations for household-level indicators varies due to missing data on number of residents reported and missing entomological data at surveys conducted prior to start of the seroconversion interval. Cross-sectional indices were calculated from entomological data observed within the seroconversion interval.

^b^ Sample size of serological observations for block-level analysis does not change as these are aggregated measures summarized over a group of households, but the number of households contributing survey data for any single block survey visit varies.

**Table 4:** Distribution of categorical indicators for serological observations

		Cross-sectional			Within 12 months	
	Number positive	Total	(%)	Number positive	Total	(%)
*Household level*						
Any adult *Ae*. *aegypti*	1924	8153	23.6	3543	8153	43.5
Any adult female *Ae*. *aegypti*	1319	8153	16.2	2578	8153	31.6
Any adult *Ae*. *aegypti* indoors	1829	8153	22.4	3364	8153	41.3
Any adult female *Ae*. *aegypti* indoors	1249	8153	15.3	2432	8153	29.8
Single Larval Method	1143	8153	14.0	2546	8145	31.2
Any pupae in household containers	610	8153	7.5	1589	8148	19.5
Container Index	1146	8153	14.1	2548	8153	31.3
*Stegomyia* Index	1146	8153	14.1	2537	8153	31.1
*Block level*						
Breteau Index	5699	7574	75.2	6713	7574	88.6
House Index	5543	7574	73.2	6451	7574	85.2
Adult Premise Index	5976	7574	78.9	6690	7574	88.4
Adult Density Index	6464	7574	85.3	7198	7574	95.1
Pupa Index	5245	7574	69.3	6729	7574	88.9
Infested Receptacle Index	6404	7574	84.6	7219	7574	95.3
Container Index	3603	7574	47.6	4426	7574	58.4
Potential Container Index	5129	7574	67.7	6424	7574	84.8
*Stegomyia* Index	6148	7574	81.2	6924	7574	91.4

### Cross-sectional densities

The adjusted RR point estimates and 95% CI are presented in Table 5. The household-level point estimates ranged from 0.75 (95% CI: 0.48, 1.34) to 1.05 (95% CI: 0.91, 1.21), suggesting no difference in the 6-month risk of DENV seroconversion based on *Ae*. *aegypti* density. At the block level, six indicators showed significant protective effects, which could be the result of higher background immunity, correlation with factors related to lower DENV risk, or chance. Compared to the adjusted RR estimates, crude risk ratio point estimates were similar for the household-level indicators and were slightly larger for block-level indicators (S1 Table).

**Table 5:** Adjusted risk ratios: association between *Ae*. *aegypti* and DENV seroconversion

	Cross-sectional Measure			Longitudinal Measure		
Indicator	Risk Ratio	95% CI		Risk Ratio	95% CI	
*Household level**^a^*						
Adult *Ae*. *aegypti* (continuous)	1.00	0.99	1.01	1.02	1.00	1.05
Any adult *Ae*. *aegypti* (categorical)	1.02	0.90	1.15	1.25	1.12	1.39
Adult female *Ae*. *aegypti* (continuous)	0.99	0.97	1.02	1.04	1.00	1.09
Any adult female *Ae*. *aegypti* (categorical)	1.03	0.90	1.18	1.29	1.16	1.44
Any adult *Ae*. *aegypti* indoors (categorical)	1.04	0.92	1.18	1.26	1.13	1.40
Any adult female *Ae*. *aegypti* indoors (categorical)	1.05	0.91	1.21	1.30	1.17	1.46
Single Larval Method (continuous)	0.97	0.88	1.06	1.07	0.98	1.16
Single Larval Method (categorical)	0.92	0.79	1.08	1.23	1.11	1.38
Pupae in household containers (continuous)	0.99	0.99	1.00	1.00	1.00	1.00
Any pupae in household containers (categorical)	0.96	0.78	1.18	1.21	1.07	1.37
Pupae per Hectare (continuous)	1.00	1.00	1.00	1.00	1.00	1.00
Pupae per Person (continuous)	0.96	0.92	1.01	1.00	0.98	1.02
Container Index (continuous)	1.00	1.00	1.00	0.98	0.95	1.00
Container Index (categorical)	0.92	0.78	1.08	1.23	1.11	1.38
*Stegomyia* Index (continuous)	0.75	0.42	1.34	1.06	0.61	1.82
*Stegomyia* Index (categorical)	0.92	0.78	1.08	1.24	1.11	1.38
*Block level**^b^*						
Breteau Index (continuous)	1.00	0.99	1.00	1.00	1.00	1.00
Breteau Index (categorical)	1.03	0.91	1.17	0.89	0.76	1.05
House Index (continuous)	0.99	0.99	1.00	1.00	0.99	1.00
House Index (categorical)	1.04	0.92	1.17	0.91	0.79	1.00
Adult Premise Index (continuous)	1.00	0.99	1.00	1.01	1.01	1.02
Adult Premise Index (categorical)	0.87	0.76	0.98	1.24	1.01	1.48
Adult Density Index (continuous)	0.96	0.84	1.10	1.24	1.02	1.50
Adult Density Index (categorical)	0.83	0.72	0.95	1.72	1.22	2.43
Pupa Index (continuous)	1.00	1.00	1.00	1.00	1.00	1.00
Pupa Index (categorical)	1.00	0.89	1.12	1.30	1.08	1.57
Pupae per Hectare (continuous)	1.00	1.00	1.00	1.00	1.00	1.00
Pupae per Person (continuous)	0.78	0.65	0.87	0.98	0.88	1.10
Infested Receptacle Index (continuous)	0.62	0.46	0.82	0.93	0.72	1.20
Infested Receptacle Index (categorical)	0.98	0.85	1.14	1.75	1.23	2.50
Container Index (continuous)	0.99	0.98	1.00	1.01	0.99	1.02
Container Index (categorical)	0.96	0.86	1.07	1.00	0.90	1.11
Potential Container Index (continuous)	0.91	0.87	0.96	1.01	1.00	1.03
Potential Container Index (categorical)	0.76	0.67	0.85	0.99	0.86	1.15
*Stegomyia* Index (continuous)	1.00	1.00	1.00	1.00	1.00	1.00
*Stegomyia* Index (categorical)	0.99	0.86	1.13	1.13	0.93	1.39

^a^Adjustment variables: DENV transmission season (May-Aug, reference group (ref); Sept-Dec, Jan-Apr); participant sex (Male; Female, ref); Participant Age (<18 years, ref; ≥18 years).

^b^Adjustment variables: Season; reported use of larvacide (yes; no, ref); participant age.

### Impact of repeated measures on household-level indicators

Using the average of densities measured in the 12 months prior to the paired sample, the RR point estimate shifted above the null for categorical measures of adult density, adult female mosquitoes, and presence of adult mosquitoes indoors (any adults as well as only females), ranging from 1.25 (95% CI: 1.12, 1.39) to 1.30 (95% CI: 1.17, 1.46). This suggests that the observation of an adult female mosquito during a household survey performed during the 12 month period prior to collection of paired sera is associated with an approximately 25% increased risk in acquisition of DENV infection compared to the risk among individuals residing in households where no adult female was observed at any survey during the 12 months preceding the paired sera. In addition, four immature stage indicators suggested an elevated risk of DENV infection: any pupae observed; the Single Larval Method (categorical); Container Index (categorical) and the *Stegomyia* Index (categorical).

### Impact of repeated measures on block-level indicators

**Fig 3:**
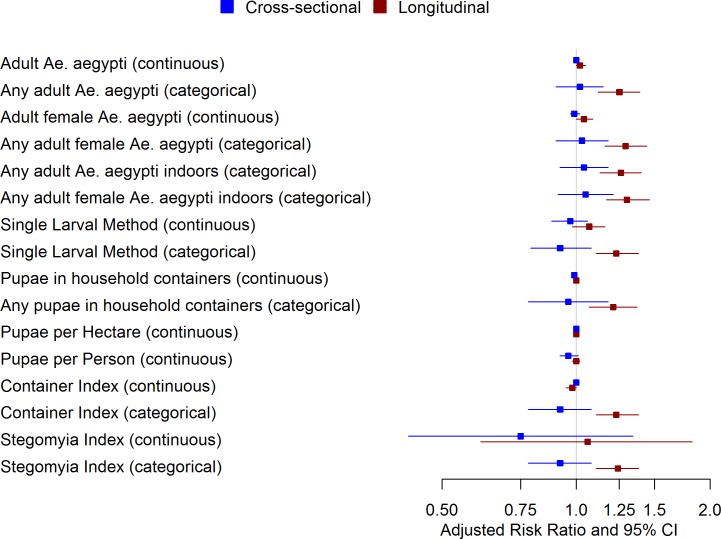
Forest plot of household-level indicators of *Ae*. *aegypti* abundance. Fig 3 compares the adjusted risk ratio and 95% CI for household-level indicators of vector density for the cross-sectional and longitudinal scenarios.

**Fig 4:**
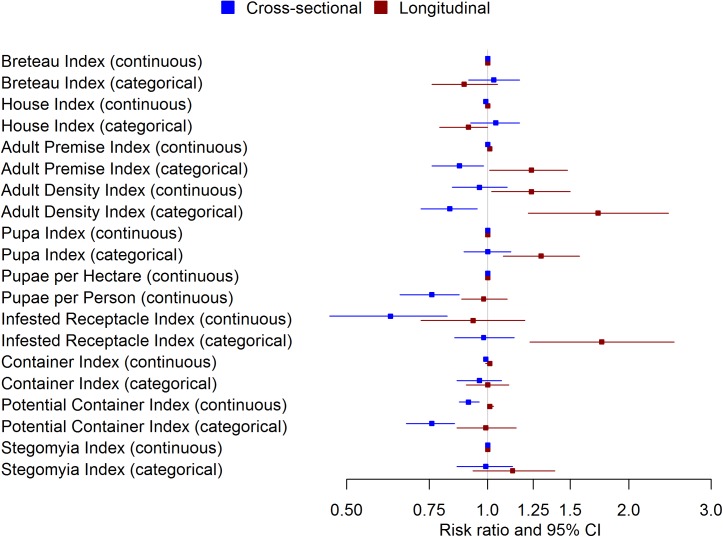
Forest plot of block-level indicators of *Ae*. *aegypti* abundance. In Figure 4, the adjusted risk ratio and 95% CI for block-level indicators of vector density for the cross-sectional and longitudinal scenarios are compared.

Analysis of block-level indicators that incorporated repeated measures demonstrated a similar trend in which all measures calculated based on adult mosquito data shifted in comparison to the cross-sectional analysis: the Adult Premise Index (RR: 1.01; 95% CI: 1.01, 1.02 when continuous and RR: 1.24; 95% CI: 1.01, 1.48 as categorical) and the Adult Density Index (RR: 1.24; 95% CI: 1.02, 1.50 as continuous and RR: 1.72; 95% CI: 1.22, 2.43 as categorical). The Pupa Index (categorical) and the Infested Receptacle Index (categorical) were the only immature stage block-level indicators to demonstrate any association with DENV infection. Figs 3 and 4 compare the risk ratios calculated for cross-sectional to longitudinal densities for both household and block-level indicators.

### Sensitivity analyses

A number of sensitivity analyses were performed to determine if the construction of the analytical cohort introduced bias. *Ae*. *aegypti* densities calculated from 6 months prior to the start of a seroconversion interval followed a similar pattern as results presented in Table 5 (S2 Table). Sensitivity analyses in which the inclusion of seroconversion events was relaxed and restricted did not alter interpretation of the main findings (S1–S3 Figs, S3–S5 Tables). Future comparison of relaxed serological inclusion criteria were compared with the 6 and 12 month longitudinal entomological measures of *Ae*. *aegypti* (S4–S6 Figs). When analyzed separately, use of different aspirators over the course of data collection did not result in substantially different results for adult stage measures (S6 Table).

## Discussion

The principal finding of this analysis is that a higher household level *Ae*. *aegypti* density calculated from cross-sectional entomological data was not associated with an increase in the risk of DENV infection. Compared to cross-sectional measures, the average *Ae*. *aegypti* density in the past 12 months resulted in more plausible effect estimates, especially for adult indices which monitor the life stage relevant to DENV transmission. Entomological evidence suggests that *Ae*. *aegypti* populations in Iquitos are highly variable in time and space [37] and the indices obtained from trimestral surveys are unlikely to capture all of the fine-scale temporal variation that occurred. The lack of an association between cross-sectional measures of larval, pupal and adult stage indicators of *Ae*. *aegypti* abundance suggests that measures of entomological risk calculated from periodic household surveys are not sufficient proxies for the 6-month risk of DENV infection. The lack of association may be the result of cross-sectional entomological survey procedures in which adult data are measured over a short period of time, resulting in lower or higher densities being attributed to the entire risk period [38]. By comparing measures of *Ae*. *aegypti* density calculated from cross-sectional data to an average density, we are able to explore the potential for non-differential measurement error of mosquito abundance to bias the association between *Ae*. *aegypti* monitoring indicators and DENV infection towards the null. This may be due to the large proportion of households with low levels of infestation being misclassified as having no *Ae*. *aegypti* present when relying on a single measurement.

Immature stage indicators were not associated with risk of DENV infection, with the exception of a few categorical indicators calculated from longitudinal data. This could be due to high larval mortality, the short lifespan of larvae and pupae, and brief time interval of data collection, resulting in immature population measures that do not always correlate in space and time with the biologically relevant adult measures [39]. For block-level indicators, aggregating household data could skew calculation of the indicator if the distribution of larval and pupal counts was concentrated in only a few households. Block-level indicators such as the Breteau Index and the House Index, which classify containers or households as “infested” if any larvae or pupae are observed, may not capture the contribution of container productivity. The pupae per person and pupae per hectare measures are sensitive to bias from inaccuracies in population or area data, as well as sampling error as the pupal life stage is ephemeral [40]. We also compared the number of infested containers at enrollment to the number observed in subsequent study visits to confirm that long-term participation in the study was not associated with improved household container management (data not presented). Prior studies also show that the spatial distribution of these measures in Iquitos varied considerably over time [37, 39].

### Strengths

The major strengths of this analysis include the use of DENV infection (not disease) as an outcome, examination of longitudinal data, and its generalizability to similar settings in which routine, periodic entomological surveillance is conducted. While dengue disease is relevant from a public health perspective and easier to quantify, DENV infection, measured as seroconversion, is more important in terms of understanding patterns of transmission from mosquitoes to humans. Most prior studies of entomological indicators and dengue outcomes [7] used symptomatic disease as the outcome. Symptomatic cases represent the small fraction of all infections that were severe enough to seek medical evaluation, thus introducing selection bias. This analysis also benefitted from longitudinal serological data, which enabled exclusion of paired sample observations once an individual was determined to no longer be at risk of infection by circulating serotypes.

Most prior studies used cross-sectional entomological data to test for an association with dengue outcomes. Longitudinal entomological monitoring allowed the use of multiple (1 to 3 per household) mosquito measures per household. This may overcome some of the measurement error of entomological assessment and account for the temporal variability associated with entomological data collection, in which a household with lower levels of abundance could be misclassified as “unexposed” to *Ae*. *aegypti*. Our results comparing the RRs estimated from cross-sectional to longitudinal entomological measures of *Ae*. *aegypti* abundance suggest the possibility that in any single entomological survey, a household with low-levels of *Ae*. *aegypti* infestation may be misclassified as having no infestation, at least for adult stage measures of abundance, which would bias the RR downwards.

The objective of this analysis was to evaluate the utility of periodic entomological monitoring as a proxy for DENV infection risk as it is typically implemented in the control setting. Under this monitoring framework, our findings are likely generalizable to similar dengue-endemic settings as the timing of serological and entomological collection employed are representative of the routine periodic monitoring used in dengue control programs. Our data have an added advantage given they were generated as part of a research study, and were subjected to rigorous monitoring of field collection procedures.

### Limitations

Our results should be interpreted in light of several limitations. First, a large proportion (9,739 of 20,176) of serological data failed to meet the 6-month inclusion criteria, which could have resulted in bias due to their exclusion. Results from sensitivity analyses to include paired samples taken more than six-months apart did not qualitatively change our findings (S1–S3 Figs). Second, the entomological and serological monitoring data relevant for DENV transmission did not perfectly coincide temporally, possibly leading to bias due to time of measurement. In sensitivity analyses, results were not sensitive to different approaches to link entomology and serology (S1–S6 Figs). Our dataset contains more entomological monitoring data than would be available in most control settings. Even with a detailed longitudinal dataset of domestic vector density, we did not observe informative associations with DENV risk. Therefore, our study reveals the inherent limitations of using *Aedes* survey methods. The design of any entomological surveillance system should consider operational feasibility given the investment needed to generate sufficient data to describe temporal and spatial variability in vector density. While ovitraps were not used in the parent studies, we expect ovitrap-based sampling of *Ae*. *aegypti* to still be subject to the same limitations that apply to monitoring pupal, larval and adult mosquitoes. Alternatives that need to be evaluated include fixed trap methods, such as ovitraps or adult trapping methods that can monitor larger areas continuously overtime, better capturing temporal differences. Monitoring traps still presents operational challenges. Methods using fixed traps usually sample fewer houses than are possible with household-based survey methods [41]. Novel technologies that capture house-to-house temporal differences are yet to be developed.

Second, even though the use of averages is not the most sophisticated method to incorporate temporal lags, it is implementable in basic statistical software and may be of utility to dengue program managers. Furthermore, in the control setting, it is highly unlikely that DENV infection outcomes would be well-resolved in time with entomological surveillance data as DENV infection cannot be monitored in real-time. Our study reports the relative risk of DENV seroconversion in a six-month period, an outcome similar in length to periods of increased dengue activity that occur seasonally in many endemic settings. While the temporal resolution between entomological and serological data collection in our study was not well resolved, our results provide quantitative evidence to challenge the use of periodic *Ae*. *aegypti* surveillance to generate suitable surrogates for DENV risk.

Third, while PRNT_70_ is the most specific serological test for dengue infection, results from this assay may be biased due to cross-reactions from antibodies directed against multiple serotypes present in a single sample or with closely related viruses. The algorithm used to classify seroconversions was conservative, possibly underestimating the number of seroconversions, but this bias is likely non-differential with respect to mosquito density. We also acknowledge the possibility that block or neighborhood-level susceptibility to DENV may affect the performance of *Ae*. *aegypti* indicators, but it was not possible to address it in the analysis without full enumeration and serological testing of the entire study population, not just sampled individuals. Nevertheless, in the endemic setting, an assumption that herd immunity exists would only further undermine the utility of entomological monitoring endpoints to serve as correlates of infection. To ensure individuals in our analysis were susceptible to DENV infection, we reviewed longitudinal serological profiles to exclude those who were likely no longer at risk of the circulating serotype. We also tested a household-level variable estimating the proportion of susceptible individuals but this had no impact on the overall results (data not presented). While our results may be generalizable to other areas with endemic transmission, this analysis should be repeated in a setting with a largely susceptible population to determine if household-based entomological surveillance is associated with DENV risk in such locations. Nevertheless, the majority of infections in our dataset were DENV3 and DENV4, which were novel at the time of introduction in the community, so herd immunity may not have played a significant role for a large subset of our data.

In this analysis, continuous indicators were tested as linear terms to maintain consistency with their definitions in the literature. It is possible that log-transformation or inclusion of polynomial terms could improve model fit, but such manipulation would reduce interpretability. For continuous indicators, the RRs measure the relative risk for a one-unit change in the indicator value; these measures are likely not informative for targeting interventions. From a public health perspective, categorical indicators are more useful to trigger vector control activities. In Iquitos, levels of infestation were heavily dispersed and binary classification (any v. none) was most informative.

Finally, it is possible that vector control efforts could have reduced the vector population, making it difficult to detect an association between *Ae*. *aegypti* density and DENV risk. Over the period included in this analysis, there were large scale vector control interventions from October 2002-Jan 2003 and others in late 2003 [42]. Nevertheless, the majority of our data included periods where there was not extensive vector control. We did control for household larvacide use as a covariate in the analysis of block-level indicators to account for the possibility that some households implemented some form of vector control.

### Conclusions

Our results provide the first quantitative evidence of the limited utility of *Ae*. *aegypti* monitoring indicators as proxy measures of DENV infection. DENV transmission is complex and time-varying; the relationship between vector density and risk is not static nor adequately characterized through periodic entomological surveillance. None of the RRs presented in this analysis represent a causal relationship between household or block-level mosquito density and true exposure to DENV. It is logistically impossible to monitor human-vector contact to establish where and when mosquito-human interaction and infection occurs. Therefore, *Ae*. *aegypti* indicators serve as surrogates of true exposure, which will always remain unmeasured. Although adult measures that incorporated longitudinal data demonstrated an association with DENV seroconversion in our study, it is possible that some unmeasured variable associated with social network patterns, housing quality and day-time human movement further modifies dengue risk.

Entomological monitoring indicators were not designed to account for the complexity of human-vector interaction, particularly given the role human movement may extend the boundaries of contact; it is likely that a substantial proportion of transmission occurs outside the home [18]. Technological advances in mosquito monitoring may eventually enable dengue control programs to quantify fluctuations in mosquito populations with greater precision across time and space. Nevertheless, DENV infection is difficulty to identify in real-time, especially given that most infections are inapparent in endemic settings. Without information on where and when individuals are infected, even detailed data of domestic vector density will require aggregation or categorization in order to attribute mosquito density to an interval-defined outcome (such as the six-month seroconversion window, as in this analysis) as DENV infection is measured at a coarse temporal interval.

Globally, the incidence of dengue has continued to intensify and expand despite significant investments in vector control [1, 43]. While vector control remains the only prevention strategy available to reduce DENV transmission in most settings, the persistence of DENV suggests transmission dynamics require a more complex understanding of human-vector interaction. Entomological monitoring will continue to serve a role in the evaluation of vector control interventions as it will be necessary to compare entomological measures of risk pre- and post-intervention as indicators of impact. Our analysis challenges the validity of most *Ae*. *aegypti* indicators as adequate proxies for true DENV exposure risk, and challenges the assumption that domestic vector data correlate with DENV transmission. In dengue-endemic settings such as Iquitos, single cross-sectional measures of adult mosquito density and the immature stage indicators commonly used by dengue control programs, such as the Breteau Index and Container Index, will likely fail to predict risk of DENV infection. Measuring adult mosquito density over multiple occasions may be the best option, but is difficult to implement. Our findings should be considered in the development and revision of enhanced DENV surveillance guidelines. Dengue control programs weighing the operational feasibility and cost of entomological monitoring against the limited utility of these indicators may wish to seek alternative monitoring frameworks that incorporate human dengue-related outcomes, such as passive case detection, and where possible, sero-surveys and active case detection.

## Supporting information

S1 STROBE Checklist (DOC)

S1 Fig Sensitivity analysis of cross-sectional household level indicators including paired serology samples taken 335–395 days apart. Risk ratios and 95% CI for an analytical dataset in which any serological paired sample taken within 335–395 days apart was split into two six-month intervals, then included in the analysis (in red). If a seroconversion occurred during that interval, it was assigned to the first six-month interval. Cross-sectional entomological data was matched to serological data by using the entomological data collected closest to the end of the paired sample interval (if there was >1 entomological measure observed within the paired sample interval). The results from the main analysis (in blue) are presented for comparison.
(PNG)

S2 Fig Sensitivity analysis of cross-sectional household level indicators including paired serology samples taken 335–395 days apart. Risk ratios and 95% CI for an analytical dataset in which any serological paired sample taken within 335–395 days apart was split into two six-month intervals, then included in the analysis (in red). If a seroconversion occurred during that interval, it was assigned to the second six-month interval. Cross-sectional entomological data was matched to serological data by using the entomological data collected closest to the end of the paired sample interval (if there was >1 entomological measure observed within the paired sample interval). The results from the main analysis (in blue) are presented for comparison.
(PNG)

S3 Fig Sensitivity analysis of cross-sectional household level indicators including paired serology samples taken 210–335 days apart. Risk ratios and 95% CI for an analytical dataset in which any serological paired sample taken within 210–335 days apart that was originally excluded from the analysis was included based on the range of dates coinciding with the annual estimated epidemic curve as described in Stoddard *et al* (2014 PLoS NTDs), then included in the analysis (in red). Cross-sectional entomological data was matched to serological data by using the entomological data collected closest to the end of the paired sample interval (if there was >1 entomological measure observed within the paired sample interval). The results from the main analysis (in blue) are presented for comparison.
(PNG)

S4 Fig Sensitivity analysis of longitudinal household level indicators including paired serology samples taken 335–395 days apart and longitudinal measures taken from prior 6 and 12 months. Risk ratios and 95% CI for an analytical dataset in which any serological paired sample taken within 335–395 days apart was split into two six-month intervals, then included in the analysis. If a seroconversion occurred during that interval, it was assigned to the first six-month interval. Longitudinal entomological data was matched to serological data by averaging observations within 6 (in red) and 12 months (in blue) preceding the seroconversion interval. The results from the main analysis (in green) are presented for comparison.
(PNG)

S5 Fig Sensitivity analysis of longitudinal household level indicators including paired serology samples taken 335–395 days apart and longitudinal measures taken from prior 6 and 12 months. Risk ratios and 95% CI for an analytical dataset in which any serological paired sample taken within 335–395 days apart was split into two six-month intervals, then included in the analysis. If a seroconversion occurred during that interval, it was assigned to the second six-month interval. Longitudinal entomological data was matched to serological data by averaging observations within 6 (in red) and 12 months (in blue) preceding the seroconversion interval. The results from the main analysis (in green) are presented for comparison.
(PNG)

S6 Fig Sensitivity analysis of longitudinal household level indicators including paired serology samples taken 210–335 days apart and longitudinal measures taken from prior 6 and 12 months. Risk ratios and 95% CI in which any serological paired sample taken within 210–335 days apart that was originally excluded from the analysis was included based on the range of dates coinciding with the annual estimated epidemic curve as described in Stoddard *et al* (2014 PLoS NTDs), then included in the analysis. Longitudinal entomological data was matched to serological data by averaging observations within 6 (in red) and 12 months (in blue) preceding the seroconversion interval. The results from the main analysis (in green) are presented for comparison.
(PNG)

S1 Table Crude RR and 95% CI. Table of crude risk ratios (RR) and 95% confidence intervals (CI) for comparison with Table 4 presented in the main analysis.
(DOCX)

S2 Table Comparison of time period for longitudinal entomological measures. Comparison of indicators calculated by averaging entomological data collected 6 and 12 months from start of seroconversion interval.
(DOCX)

S3 Table Adjusted RRs and 95% CI for inclusive serological criteria. Adjusted risk ratios (RR) and 95% confidence intervals (CI) in which any positive serological result was classified as a seroconversion event compared to the RR and 95% CI presented in the main analysis in which discrepant longitudinal serological samples were excluded.
(DOCX)

S4 Table Adjusted RRs and 95% CI for more restrictive serological criteria. Adjusted risk ratios (RR) and 95% confidence intervals (CI) in which any serological result that tested positive for more than one serotype in the same paired sample was excluded compared to the RR and 95% CI presented in the main analysis in which these serological samples were included.
(DOCX)

S5 Table Adjusted RRs and 95% CI for more restrictive serological criteria, excluding any serological result with evidence of prior DENV1 or DENV2 infection. Adjusted risk ratios (RR) and 95% confidence intervals (CI) in which any serological sample collected between January 2001-December 2003 had prior evidence of DENV1 or DENV2 infection was excluded compared to the RR and 95% CI presented in the main analysis in which these serological samples were included.
(DOCX)

S6 Table Adjusted RRs and 95% CI stratified by aspirator type. Adjusted risk ratios (RR) and 95% confidence intervals (CI) for cross-sectional adult stage measures of *Ae*. *aegypti* comparing data collected during periods in which the aspirator was changed in 2009: 1999–2008, the CDC backpack aspirator and 2010, the Prokopack aspirator.
(DOCX)
