# Supplementary figures and images for: The relationship between entomological indicators of Aedes aegypti abundance and dengue virus infection
Source: PLoS Negl Trop Dis. 2017 Mar 23;11(3):e0005429. doi: 10.1371/journal.pntd.0005429 (PMC5363802; doi:10.1371/journal.pntd.0005429)

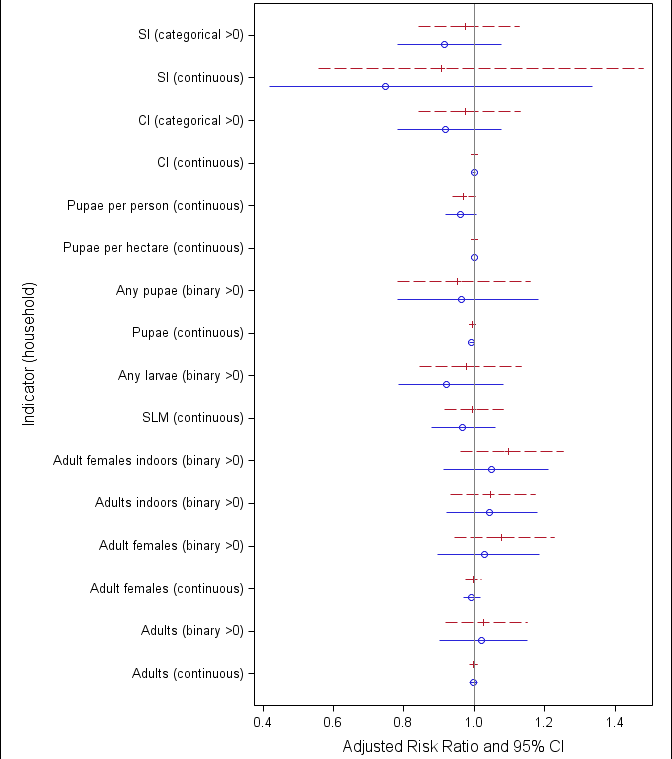

Supplement: S1 Fig — Risk ratios and 95% CI for an analytical dataset in which any serological paired sample taken within 335–395 days apart was split into two six-month intervals, then included in the analysis (in red). If a seroconversion occurred during that interval, it was assigned to the first six-month interval. Cross-sectional entomological data was matched to serological data by using the entomological data collected closest to the end of the paired sample interval (if there was >1 entomological measure observed within the paired sample interval). The results from the main analysis (in blue) are presented for comparison. (PNG) [file pntd.0005429.s002.png]

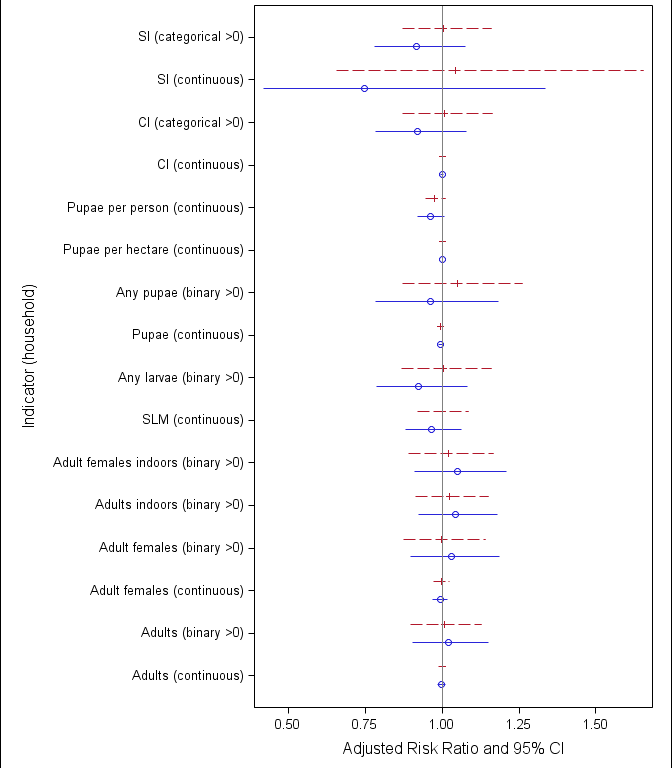

Supplement: S2 Fig — Risk ratios and 95% CI for an analytical dataset in which any serological paired sample taken within 335–395 days apart was split into two six-month intervals, then included in the analysis (in red). If a seroconversion occurred during that interval, it was assigned to the second six-month interval. Cross-sectional entomological data was matched to serological data by using the entomological data collected closest to the end of the paired sample interval (if there was >1 entomological measure observed within the paired sample interval). The results from the main analysis (in blue) are presented for comparison. (PNG) [file pntd.0005429.s003.png]

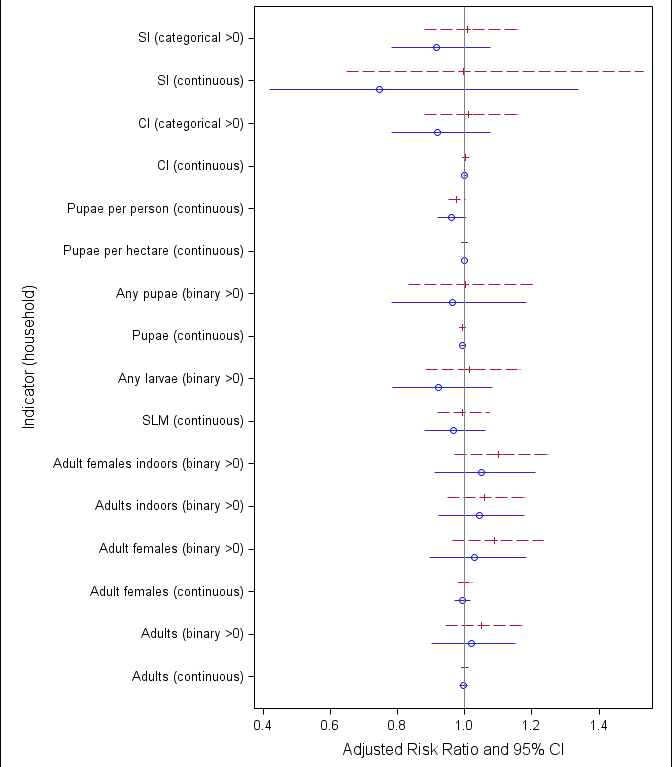

Supplement: S3 Fig — Risk ratios and 95% CI for an analytical dataset in which any serological paired sample taken within 210–335 days apart that was originally excluded from the analysis was included based on the range of dates coinciding with the annual estimated epidemic curve as described in Stoddard et al (2014 PLoS NTDs), then included in the analysis (in red). Cross-sectional entomological data was matched to serological data by using the entomological data collected closest to the end of the paired sample interval (if there was >1 entomological measure observed within the paired sample interval). The results from the main analysis (in blue) are presented for comparison. (PNG) [file pntd.0005429.s004.png]

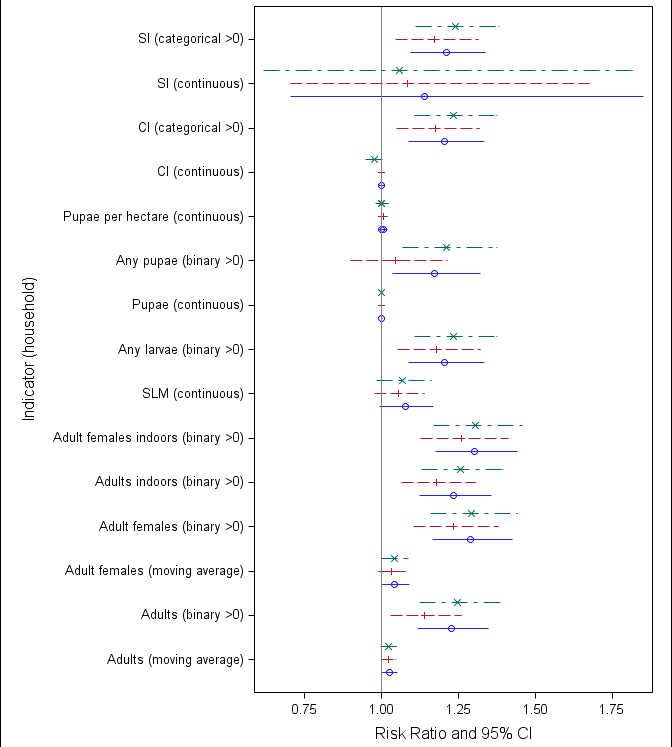

Supplement: S4 Fig — Risk ratios and 95% CI for an analytical dataset in which any serological paired sample taken within 335–395 days apart was split into two six-month intervals, then included in the analysis. If a seroconversion occurred during that interval, it was assigned to the first six-month interval. Longitudinal entomological data was matched to serological data by averaging observations within 6 (in red) and 12 months (in blue) preceding the seroconversion interval. The results from the main analysis (in green) are presented for comparison. (PNG) [file pntd.0005429.s005.png]

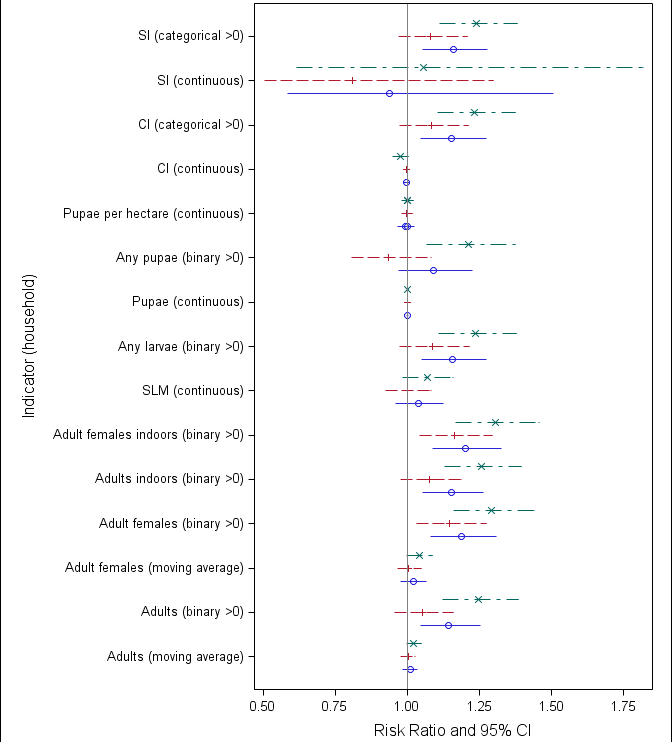

Supplement: S5 Fig — Risk ratios and 95% CI for an analytical dataset in which any serological paired sample taken within 335–395 days apart was split into two six-month intervals, then included in the analysis. If a seroconversion occurred during that interval, it was assigned to the second six-month interval. Longitudinal entomological data was matched to serological data by averaging observations within 6 (in red) and 12 months (in blue) preceding the seroconversion interval. The results from the main analysis (in green) are presented for comparison. (PNG) [file pntd.0005429.s006.png]

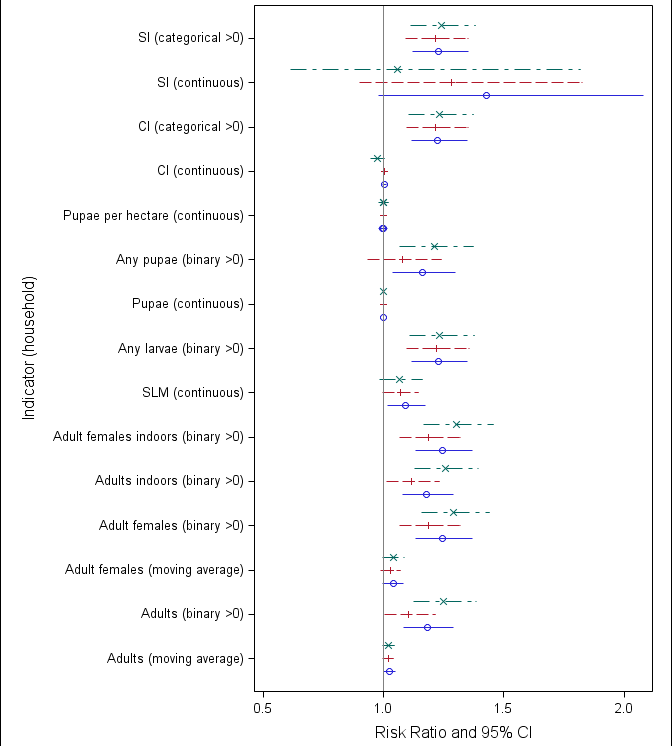

Supplement: S6 Fig — Risk ratios and 95% CI in which any serological paired sample taken within 210–335 days apart that was originally excluded from the analysis was included based on the range of dates coinciding with the annual estimated epidemic curve as described in Stoddard et al (2014 PLoS NTDs), then included in the analysis. Longitudinal entomological data was matched to serological data by averaging observations within 6 (in red) and 12 months (in blue) preceding the seroconversion interval. The results from the main analysis (in green) are presented for comparison. (PNG) [file pntd.0005429.s007.png]
