# Supplementary material for: The relationship between entomological indicators of Aedes aegypti abundance and dengue virus infection
Source: PLoS Negl Trop Dis. 2017 Mar 23;11(3):e0005429. doi: 10.1371/journal.pntd.0005429 (PMC5363802; doi:10.1371/journal.pntd.0005429)
Supplement: S1 Table — Table of crude risk ratios (RR) and 95% confidence intervals (CI) for comparison with Table 4 presented in the main analysis. (DOCX) [file pntd.0005429.s008.docx]

|  |  | **Cross-sectional** | | |  | **Longitudinal** | | |
| --- | --- | --- | --- | --- | --- | --- | --- | --- |
| **Indicator** |  | **Risk Ratio** | **95% CI** | |  | **Risk Ratio** | **95% CI** | |
| *Household level* |  |  |  |  |  |  |  |  |
| Adult *Ae. aegypti* (continuous) |  | 1.00 | 0.99 | 1.01 |  | 1.02 | 0.99 | 1.04 |
| Any adult *Ae. aegypti* (categorical) |  | 1.03 | 0.91 | 1.16 |  | 1.23 | 1.11 | 1.37 |
| Adult female *Ae. aegypti* (continuous) |  | 0.99 | 0.97 | 1.02 |  | 1.04 | 0.99 | 1.08 |
| Any adult female *Ae. aegypti* (categorical) |  | 1.04 | 0.90 | 1.19 |  | 1.26 | 1.13 | 1.41 |
| Any adult *Ae. aegypti* indoors (categorical) |  | 1.06 | 0.93 | 1.19 |  | 1.25 | 1.12 | 1.39 |
| Any adult female *Ae. aegypti* indoors (categorical) | | 1.06 | 0.92 | 1.22 |  | 1.28 | 1.15 | 1.43 |
| Single Larval Method (continuous) |  | 0.94 | 0.85 | 1.04 |  | 1.03 | 0.95 | 1.13 |
| Single Larval Method (categorical) |  | 0.88 | 0.75 | 1.03 |  | 1.18 | 1.06 | 1.31 |
| Pupae in household containers (continuous) |  | 0.99 | 0.98 | 1.00 |  | 1.00 | 1.00 | 1.01 |
| Any pupae in household containers (categorical) |  | 0.91 | 0.74 | 1.13 |  | 1.14 | 1.01 | 1.30 |
| Pupae per Hectare (continuous) |  | 1.00 | 1.00 | 1.00 |  | 1.00 | 1.00 | 1.00 |
| Pupae per Person (continuous) |  | 0.96 | 0.91 | 1.01 |  | 1.00 | 0.98 | 1.03 |
| Container Index (continuous) |  | 1.00 | 0.99 | 1.00 |  | 0.97 | 0.95 | 1.00 |
| Container Index (categorical) |  | 0.87 | 0.75 | 1.03 |  | 1.18 | 1.06 | 1.31 |
| *Stegomyia* Index (continuous) |  | 0.68 | 0.37 | 1.25 |  | 0.92 | 0.52 | 1.64 |
| *Stegomyia* Index (categorical) |  | 0.87 | 0.74 | 1.02 |  | 1.18 | 1.06 | 1.32 |
|  |  |  |  |  |  |  |  |  |
| *Block level* |  |  |  |  |  |  |  |  |
| Breteau Index (continuous) |  | 0.99 | 0.99 | 0.99 |  | 1.00 | 0.99 | 1.00 |
| Breteau Index (categorical) |  | 0.95 | 0.84 | 1.07 |  | 0.93 | 0.79 | 1.09 |
| House Index (continuous) |  | 0.99 | 0.98 | 0.99 |  | 0.99 | 0.99 | 1.00 |
| House Index (categorical) |  | 0.93 | 0.83 | 1.05 |  | 0.89 | 0.78 | 1.03 |
| Adult Premise Index (continuous) |  | 1.00 | 0.99 | 1.00 |  | 1.01 | 1.00 | 1.01 |
| Adult Premise Index (categorical) |  | 0.87 | 0.77 | 0.98 |  | 1.29 | 1.06 | 1.56 |
| Adult Density Index (continuous) |  | 0.96 | 0.85 | 1.09 |  | 1.13 | 0.93 | 1.38 |
| Adult Density Index (categorical) |  | 0.82 | 0.72 | 0.95 |  | 1.91 | 1.35 | 2.71 |
| Pupa Index (continuous) |  | 1.00 | 1.00 | 1.00 |  | 1.00 | 1.00 | 1.00 |
| Pupa Index (categorical) |  | 0.91 | 0.81 | 1.02 |  | 1.32 | 1.09 | 1.60 |
| Pupae per Hectare (continuous) |  | 1.00 | 1.00 | 1.00 |  | 1.00 | 1.00 | 1.00 |
| Pupae per Person (continuous) |  | 0.69 | 0.58 | 0.81 |  | 1.00 | 1.00 | 1.00 |
| Infested Receptacle Index (continuous) |  | 0.45 | 0.34 | 0.60 |  | 0.66 | 0.51 | 0.85 |
| Infested Receptacle Index (categorical) |  | 0.98 | 0.84 | 1.13 |  | 2.09 | 1.47 | 2.98 |
| Container Index (continuous) |  | 0.98 | 0.97 | 0.99 |  | 0.99 | 0.98 | 1.01 |
| Container Index (categorical) |  | 0.86 | 0.77 | 0.96 |  | 0.89 | 0.79 | 0.99 |
| Potential Container Index (continuous) |  | 0.86 | 0.82 | 0.90 |  | 1.00 | 0.99 | 1.02 |
| Potential Container Index (categorical) |  | 0.64 | 0.57 | 0.71 |  | 0.99 | 0.85 | 1.15 |
| *Stegomyia* Index (continuous) |  | 1.00 | 0.99 | 1.00 |  | 1.00 | 1.00 | 1.00 |
| *Stegomyia* Index (categorical) |  | 0.95 | 0.83 | 1.09 |  | 1.23 | 1.00 | 1.51 |
