# Supplementary material for: The relationship between entomological indicators of Aedes aegypti abundance and dengue virus infection
Source: PLoS Negl Trop Dis. 2017 Mar 23;11(3):e0005429. doi: 10.1371/journal.pntd.0005429 (PMC5363802; doi:10.1371/journal.pntd.0005429)
Supplement: S2 Table — Comparison of indicators calculated by averaging entomological data collected 6 and 12 months from start of seroconversion interval. (DOCX) [file pntd.0005429.s009.docx]

|  |  | **Within 12 months** | | |  | **Within 6 months** | | |
| --- | --- | --- | --- | --- | --- | --- | --- | --- |
| **Indicator** |  | **Risk Ratio** | **95% CI** | |  | **Risk Ratio** | **95% CI** | |
| *Household level* |  |  |  |  |  |  |  |  |
| Adult *Ae. aegypti* (continuous) |  | 1.02 | 1.00 | 1.05 |  | 1.02 | 0.99 | 1.04 |
| Any adult *Ae. aegypti* (categorical) |  | 1.25 | 1.12 | 1.39 |  | 1.14 | 1.02 | 1.27 |
| Adult female *Ae. aegypti* (continuous) |  | 1.04 | 1.00 | 1.09 |  | 1.02 | 0.98 | 1.07 |
| Any adult female *Ae. aegypti* (categorical) |  | 1.29 | 1.16 | 1.44 |  | 1.23 | 1.09 | 1.38 |
| Any adult *Ae.aegypti* indoors (categorical) |  | 1.26 | 1.13 | 1.40 |  | 1.18 | 1.06 | 1.32 |
| Any adult female *Ae. aegypti* indoors (categorical) |  | 1.30 | 1.17 | 1.46 |  | 1.25 | 1.11 | 1.41 |
| Single Larval Method (continuous) |  | 1.07 | 0.98 | 1.16 |  | 1.03 | 0.95 | 1.12 |
| Single Larval Method (categorical) |  | 1.23 | 1.11 | 1.38 |  | 1.16 | 1.02 | 1.31 |
| Pupae in household containers (continuous) |  | 1.00 | 1.00 | 1.00 |  | 1.00 | 1.00 | 1.00 |
| Any pupae in household containers (categorical) |  | 1.21 | 1.07 | 1.37 |  | 1.04 | 0.89 | 1.22 |
| Pupae per Hectare (continuous) |  | 1.00 | 1.00 | 1.00 |  | 1.00 | 1.00 | 1.00 |
| Pupae per Person (continuous) |  | 1.00 | 0.98 | 1.03 |  | 1.00 | 0.98 | 1.03 |
| Container Index (continuous) |  | 0.98 | 0.95 | 1.00 |  | 0.99 | 0.97 | 1.00 |
| Container Index (categorical) |  | 1.23 | 1.11 | 1.38 |  | 1.16 | 1.02 | 1.31 |
| *Stegomyia* Index (continuous) |  | 1.06 | 0.61 | 1.82 |  | 0.93 | 0.56 | 1.55 |
| *Stegomyia* Index (categorical) |  | 1.24 | 1.11 | 1.38 |  | 1.15 | 1.02 | 1.30 |
|  |  |  |  |  |  |  |  |  |
| *Block level* |  |  |  |  |  |  |  |  |
| Breteau Index (continuous) |  | 1.00 | 1.00 | 1.00 |  | 1.00 | 0.99 | 1.00 |
| Breteau Index (categorical) |  | 0.89 | 0.76 | 1.05 |  | 1.07 | 0.92 | 1.23 |
| House Index (continuous) |  | 1.00 | 0.99 | 1.00 |  | 1.00 | 0.99 | 1.00 |
| House Index (categorical) |  | 0.91 | 0.79 | 1.00 |  | 0.96 | 0.85 | 1.09 |
| Adult Premise Index (continuous) |  | 1.01 | 1.01 | 1.02 |  | 1.01 | 1.00 | 1.01 |
| Adult Premise Index (categorical) |  | 1.24 | 1.01 | 1.48 |  | 1.05 | 0.90 | 1.22 |
| Adult Density Index (continuous) |  | 1.24 | 1.02 | 1.50 |  | 1.22 | 1.04 | 1.44 |
| Adult Density Index (categorical) |  | 1.72 | 1.22 | 2.43 |  | 1.40 | 1.09 | 1.80 |
| Pupa Index (continuous) |  | 1.00 | 1.00 | 1.00 |  | 1.00 | 1.00 | 1.00 |
| Pupa Index (categorical) |  | 1.30 | 1.08 | 1.57 |  | 1.04 | 0.91 | 1.20 |
| Pupae per Hectare (continuous) |  | 1.00 | 1.00 | 1.00 |  | 1.00 | 1.00 | 1.00 |
| Pupae per Person (continuous) |  | 1.00 | 1.00 | 1.00 |  | 1.00 | 1.00 | 1.00 |
| Infested Receptacle Index (continuous) |  | 0.93 | 0.72 | 1.20 |  | 0.79 | 0.62 | 1.02 |
| Infested Receptacle Index (categorical) |  | 1.75 | 1.23 | 2.50 |  | 1.15 | 0.92 | 1.43 |
| Container Index (continuous) |  | 1.01 | 0.99 | 1.02 |  | 0.99 | 0.88 | 1.10 |
| Container Index (categorical) |  | 1.00 | 0.90 | 1.11 |  | 0.88 | 0.79 | 0.98 |
| Potential Container Index (continuous) |  | 1.01 | 1.00 | 1.03 |  | 1.00 | 0.99 | 1.01 |
| Potential Container Index (categorical) |  | 0.99 | 0.86 | 1.15 |  | 1.09 | 0.95 | 1.25 |
| *Stegomyia* Index (continuous) |  | 1.00 | 1.00 | 1.00 |  | 1.00 | 1.00 | 1.00 |
| *Stegomyia* Index (categorical) |  | 1.13 | 0.93 | 1.39 |  | 1.03 | 0.87 | 1.22 |
