# Supplementary material for: The relationship between entomological indicators of Aedes aegypti abundance and dengue virus infection
Source: PLoS Negl Trop Dis. 2017 Mar 23;11(3):e0005429. doi: 10.1371/journal.pntd.0005429 (PMC5363802; doi:10.1371/journal.pntd.0005429)
Supplement: S3 Table — Adjusted risk ratios (RR) and 95% confidence intervals (CI) in which any positive serological result was classified as a seroconversion event compared to the RR and 95% CI presented in the main analysis in which discrepant longitudinal serological samples were excluded. (DOCX) [file pntd.0005429.s010.docx]

|  |  | **Cross-sectional** | | |  | **Longitudinal** | | |
| --- | --- | --- | --- | --- | --- | --- | --- | --- |
| **Indicator** |  | **Risk Ratio** | **95% CI** | |  | **Risk Ratio** | **95% CI** | |
| *Household level* |  |  |  |  |  |  |  |  |
| Adult *Ae. aegypti* (continuous) |  | 1.00 | 0.99 | 1.01 |  | 1.00 | 0.98 | 1.03 |
| Any adult *Ae. aegypti* (categorical) |  | 1.09 | 0.98 | 1.21 |  | 1.13 | 1.03 | 1.24 |
| Adult female *Ae. aegypti* (continuous) |  | 1.00 | 0.98 | 1.02 |  | 1.00 | 0.96 | 1.05 |
| Any adult female *Ae. aegypti* (categorical) |  | 1.11 | 0.99 | 1.25 |  | 1.15 | 1.04 | 1.27 |
| Any adult *Ae. aegypti* indoors (categorical) |  | 1.11 | 1.00 | 1.23 |  | 1.14 | 1.04 | 1.25 |
| Any adult female *Ae. aegypti* indoors (categorical) | | 1.14 | 1.01 | 1.28 |  | 1.16 | 1.05 | 1.28 |
| Single Larval Method (continuous) |  | 0.92 | 0.84 | 1.01 |  | 0.97 | 0.89 | 1.06 |
| Single Larval Method (categorical) |  | 0.86 | 0.74 | 1.00 |  | 1.11 | 1.00 | 1.22 |
| Pupae in household containers (continuous) |  | 0.99 | 0.98 | 1.00 |  | 1.00 | 1.00 | 1.00 |
| Any pupae in household containers (categorical) |  | 0.86 | 0.71 | 1.05 |  | 1.09 | 0.97 | 1.22 |
| Pupae per Hectare (continuous) |  | 1.00 | 1.00 | 1.00 |  | 1.00 | 1.00 | 1.00 |
| Pupae per Person (continuous) |  | 0.94 | 0.88 | 1.00 |  | 1.00 | 0.98 | 1.02 |
| Container Index (continuous) |  | 1.00 | 1.00 | 1.00 |  | 0.97 | 0.94 | 1.00 |
| Container Index (categorical) |  | 0.86 | 0.74 | 1.00 |  | 1.10 | 1.00 | 1.22 |
| *Stegomyia* Index (continuous) |  | 0.65 | 0.37 | 1.14 |  | 0.76 | 0.42 | 1.37 |
| *Stegomyia* Index (categorical) |  | 0.86 | 0.74 | 1.00 |  | 1.11 | 1.00 | 1.22 |
|  |  |  |  |  |  |  |  |  |
| *Block level* |  |  |  |  |  |  |  |  |
| Breteau Index (continuous) |  | 0.99 | 0.99 | 0.99 |  | 0.99 | 0.99 | 1.00 |
| Breteau Index (categorical) |  | 0.96 | 0.86 | 1.07 |  | 0.78 | 0.68 | 0.89 |
| House Index (continuous) |  | 0.98 | 0.98 | 0.99 |  | 0.98 | 0.98 | 0.99 |
| House Index (categorical) |  | 0.93 | 0.84 | 1.03 |  | 0.79 | 0.71 | 0.89 |
| Adult Premise Index (continuous) |  | 1.00 | 1.00 | 1.01 |  | 0.99 | 0.99 | 1.00 |
| Adult Premise Index (categorical) |  | 1.01 | 0.90 | 1.14 |  | 1.02 | 0.88 | 1.19 |
| Adult Density Index (continuous) |  | 1.07 | 0.97 | 1.18 |  | 0.87 | 0.68 | 1.09 |
| Adult Density Index (categorical) |  | 1.00 | 0.87 | 1.14 |  | 1.48 | 1.08 | 2.03 |
| Pupa Index (continuous) |  | 1.00 | 1.00 | 1.00 |  | 1.00 | 1.00 | 1.00 |
| Pupa Index (categorical) |  | 0.92 | 0.83 | 1.01 |  | 1.10 | 0.94 | 1.29 |
| Pupae per Hectare (continuous) |  | 1.00 | 1.00 | 1.00 |  | 1.00 | 1.00 | 1.00 |
| Pupae per Person (continuous) |  | 0.89 | 0.79 | 1.01 |  | 1.00 | 1.00 | 1.00 |
| Infested Receptacle Index (continuous) |  | 0.40 | 0.30 | 0.53 |  | 0.47 | 0.35 | 0.63 |
| Infested Receptacle Index (categorical) |  | 1.01 | 0.89 | 1.15 |  | 1.61 | 1.11 | 2.33 |
| Container Index (continuous) |  | 0.98 | 0.97 | 0.99 |  | 0.98 | 0.97 | 0.99 |
| Container Index (categorical) |  | 1.01 | 0.89 | 1.15 |  | 0.81 | 0.74 | 0.89 |
| Potential Container Index (continuous) |  | 0.82 | 0.79 | 0.86 |  | 0.98 | 0.97 | 0.99 |
| Potential Container Index (categorical) |  | 0.58 | 0.53 | 0.65 |  | 0.89 | 0.79 | 1.01 |
| *Stegomyia* Index (continuous) |  | 0.99 | 0.99 | 1.00 |  | 1.00 | 0.99 | 1.00 |
| *Stegomyia* Index (categorical) |  | 1.01 | 0.89 | 1.15 |  | 0.96 | 0.81 | 1.14 |
