# Supplementary material for: The relationship between entomological indicators of Aedes aegypti abundance and dengue virus infection
Source: PLoS Negl Trop Dis. 2017 Mar 23;11(3):e0005429. doi: 10.1371/journal.pntd.0005429 (PMC5363802; doi:10.1371/journal.pntd.0005429)
Supplement: S4 Table — Adjusted risk ratios (RR) and 95% confidence intervals (CI) in which any serological result that tested positive for more than one serotype in the same paired sample was excluded compared to the RR and 95% CI presented in the main analysis in which these serological samples were included. (DOCX) [file pntd.0005429.s011.docx]

|  |  | **Cross-sectional** | | |  | **Longitudinal** | | | | |
| --- | --- | --- | --- | --- | --- | --- | --- | --- | --- | --- |
| **Indicator** |  | **Risk Ratio** | **95% CI** | |  | **Risk Ratio** | **95% CI** | | | |
| *Household level* |  |  |  |  |  |  |  | |  | |
| Adult *Ae. aegypti* (continuous) |  | 1.00 | 0.98 | 1.01 |  | 1.05 | 1.02 | | 1.08 | |
| Any adult *Ae. aegypti* (categorical) |  | 1.01 | 0.88 | 1.16 |  | 1.37 | 1.22 | | 1.55 | |
| Adult female *Ae. aegypti* (continuous) |  | 0.99 | 0.97 | 1.02 |  | Did not converge | |  | |  |
| Any adult female *Ae. aegypti* (categorical) |  | 1.04 | 0.89 | 1.22 |  | 1.36 | 1.20 | | 1.54 | |
| Any adult *Ae. aegypti* indoors (categorical) |  | 1.03 | 0.89 | 1.18 |  | 1.39 | 1.24 | | 1.57 | |
| Any adult female *Ae. aegypti* indoors (categorical) | | 1.06 | 0.90 | 1.25 |  | 1.41 | 1.24 | | 1.60 | |
| Single Larval Method (continuous) |  | 1.02 | 0.93 | 1.12 |  | 1.10 | 0.99 | | 1.21 | |
| Single Larval Method (categorical) |  | 1.04 | 0.88 | 1.24 |  | 1.15 | 1.01 | | 1.31 | |
| Pupae in household containers (continuous) |  | 0.99 | 0.99 | 1.00 |  | Did not converge | | |  | |
| Any pupae in household containers (categorical) |  | 1.07 | 0.86 | 1.34 |  | 1.21 | 1.04 | | 1.41 | |
| Pupae per Hectare (continuous) |  | 1.00 | 1.00 | 1.00 |  | 1.00 | 1.00 | | 1.00 | |
| Pupae per Person (continuous) |  | 0.97 | 0.94 | 1.01 |  | 1.00 | 1.00 | | 1.01 | |
| Container Index (continuous) |  | 0.85 | 0.46 | 1.58 |  | 1.00 | 1.00 | | 1.01 | |
| Container Index (categorical) |  | 1.00 | 1.00 | 1.01 |  | 1.15 | 1.01 | | 1.31 | |
| *Stegomyia* Index (continuous) |  | 1.00 | 0.57 | 1.76 |  | 1.23 | 0.70 | | 2.15 | |
| *Stegomyia* Index (categorical) |  | 1.04 | 0.87 | 1.23 |  | 1.15 | 1.01 | | 1.31 | |
|  |  |  |  |  |  |  |  | |  | |
| *Block level* |  |  |  |  |  |  |  | |  | |
| Breteau Index (continuous) |  | 1.00 | 0.99 | 1.00 |  | 1.00 | 1.00 | | 1.00 | |
| Breteau Index (categorical) |  | 1.00 | 0.86 | 1.15 |  | 0.95 | 0.79 | | 1.15 | |
| House Index (continuous) |  | 0.99 | 0.99 | 1.00 |  | 1.00 | 0.99 | | 1.01 | |
| House Index (categorical) |  | 1.01 | 0.88 | 1.16 |  | 0.95 | 0.80 | | 1.12 | |
| Adult Premise Index (continuous) |  | 1.00 | 0.99 | 1.00 |  | 1.01 | 1.01 | | 1.02 | |
| Adult Premise Index (categorical) |  | 0.87 | 0.75 | 1.01 |  | 1.27 | 1.03 | | 1.58 | |
| Adult Density Index (continuous) |  | 0.96 | 0.83 | 1.12 |  | 1.36 | 1.12 | | 1.67 | |
| Adult Density Index (categorical) |  | 0.88 | 0.75 | 1.05 |  | 1.48 | 1.03 | | 2.14 | |
| Pupa Index (continuous) |  | 1.00 | 1.00 | 1.00 |  | 1.00 | 1.00 | | 1.00 | |
| Pupa Index (categorical) |  | 0.98 | 0.86 | 1.13 |  | 1.32 | 1.05 | | 1.64 | |
| Pupae per Hectare (continuous) |  | 1.00 | 1.00 | 1.00 |  | 1.00 | 1.00 | | 1.00 | |
| Pupae per Person (continuous) |  | 0.96 | 0.91 | 1.01 |  | 1.00 | 1.00 | | 1.00 | |
| Infested Receptacle Index (continuous) |  | 0.65 | 0.46 | 0.91 |  | 1.07 | 0.81 | | 1.42 | |
| Infested Receptacle Index (categorical) |  | 0.95 | 0.80 | 1.12 |  | 1.88 | 1.22 | | 2.89 | |
| Container Index (continuous) |  | 0.99 | 0.97 | 1.00 |  | 1.01 | 0.99 | | 1.03 | |
| Container Index (categorical) |  | 0.95 | 0.80 | 1.12 |  | 1.04 | 0.92 | | 1.19 | |
| Potential Container Index (continuous) |  | 0.93 | 0.88 | 0.98 |  | 1.02 | 1.00 | | 1.03 | |
| Potential Container Index (categorical) |  | 0.80 | 0.70 | 0.92 |  | 1.01 | 0.85 | | 1.20 | |
| *Stegomyia* Index (continuous) |  | 1.00 | 1.00 | 1.00 |  | 1.00 | 1.00 | | 1.00 | |
| *Stegomyia* Index (categorical) |  | 0.95 | 0.80 | 1.12 |  | 1.15 | 0.91 | | 1.47 | |
