# Supplementary material for: The relationship between entomological indicators of Aedes aegypti abundance and dengue virus infection
Source: PLoS Negl Trop Dis. 2017 Mar 23;11(3):e0005429. doi: 10.1371/journal.pntd.0005429 (PMC5363802; doi:10.1371/journal.pntd.0005429)
Supplement: S5 Table — Adjusted risk ratios (RR) and 95% confidence intervals (CI) in which any serological sample collected between January 2001-December 2003 had prior evidence of DENV1 or DENV2 infection was excluded compared to the RR and 95% CI presented in the main analysis in which these serological samples were included. (DOCX) [file pntd.0005429.s012.docx]

|  |  | **Cross-sectional** | | |  | **Longitudinal** | | |
| --- | --- | --- | --- | --- | --- | --- | --- | --- |
| **Indicator** |  | **Risk Ratio** | **95% CI** | |  | **Risk Ratio** | **95% CI** | |
| *Household level* |  |  |  |  |  |  |  |  |
| Adult *Ae. aegypti* (continuous) |  | 0.82 | 0.65 | 1.04 |  | * |  |  |
| Any adult *Ae. aegypti* (categorical) |  | 0.98 | 0.52 | 1.84 |  | 1.80 | 1.90 | 2.40 |
| Adult female *Ae. aegypti* (continuous) |  | 0.94 | 0.77 | 1.16 |  | * |  |  |
| Any adult female *Ae. aegypti* (categorical) |  | 1.35 | 0.71 | 2.58 |  | 1.89 | 1.23 | 2.88 |
| Any adult *Ae. aegypti* indoors (categorical) |  | 0.90 | 0.46 | 1.77 |  | 1.71 | 1.12 | 2.60 |
| Any adult female *Ae. aegypti* indoors (categorical) | | 1.44 | 0.76 | 2.75 |  | 2.06 | 1.35 | 3.15 |
| Single Larval Method (continuous) |  | 0.85 | 0.50 | 1.45 |  | * |  |  |
| Single Larval Method (categorical) |  | 0.61 | 0.27 | 1.37 |  | 0.61 | 0.27 | 1.35 |
| Pupae in household containers (continuous) |  | 0.98 | 0.95 | 1.01 |  | * |  |  |
| Any pupae in household containers (categorical) |  | 1.08 | 0.45 | 2.60 |  | 1.67 | 1.05 | 2.64 |
| Pupae per Hectare (continuous) |  | 1.00 | 1.00 | 1.00 |  | * |  |  |
| Pupae per Person (continuous) |  | 0.95 | 0.85 | 1.06 |  | 1.23 | 1.12 | 1.30 |
| Container Index (continuous) |  | 0.98 | 0.96 | 1.00 |  | 0.97 | 0.87 | 1.07 |
| Container Index (categorical) |  | 0.61 | 0.27 | 1.35 |  | 0.60 | 0.27 | 1.33 |
| *Stegomyia* Index (continuous) |  | 0.83 | 0.07 | 9.80 |  | * |  |  |
| *Stegomyia* Index (categorical) |  | 0.61 | 0.27 | 1.35 |  | 0.60 | 0.27 | 1.33 |
|  |  |  |  |  |  |  |  |  |
| *Block level* |  |  |  |  |  |  |  |  |
| Breteau Index (continuous) |  | 0.98 | 0.97 | 1.00 |  | 0.99 | 0.98 | 1.01 |
| Breteau Index (categorical) |  | 0.69 | 0.44 | 1.10 |  | 0.83 | 0.49 | 1.39 |
| House Index (continuous) |  | 0.97 | 0.95 | 1.00 |  | 1.00 | 0.98 | 1.02 |
| House Index (categorical) |  | 0.68 | 0.43 | 1.08 |  | 0.80 | 0.49 | 1.32 |
| Adult Premise Index (continuous) |  | 0.97 | 0.95 | 1.00 |  | 1.02 | 1.00 | 1.05 |
| Adult Premise Index (categorical) |  | 0.59 | 0.37 | 0.94 |  | 1.16 | 0.53 | 2.54 |
| Adult Density Index (continuous) |  | 0.61 | 0.19 | 1.96 |  | 1.64 | 1.04 | 2.58 |
| Adult Density Index (categorical) |  | 0.82 | 0.50 | 1.33 |  | * |  |  |
| Pupa Index (continuous) |  | 1.00 | 1.00 | 1.00 |  | 1.00 | 1.00 | 1.00 |
| Pupa Index (categorical) |  | 0.72 | 0.46 | 1.13 |  | 1.01 | 0.56 | 1.81 |
| Pupae per Hectare (continuous) |  | 1.00 | 1.00 | 1.00 |  | 1.00 | 1.00 | 1.00 |
| Pupae per Person (continuous) |  | 0.96 | 0.86 | 1.07 |  | 1.00 | 1.00 | 1.00 |
| Infested Receptacle Index (continuous) |  | 0.17 | 0.04 | 0.74 |  | 0.57 | 0.20 | 1.66 |
| Infested Receptacle Index (categorical) |  | 0.63 | 0.39 | 1.01 |  | 1.22 | 0.55 | 2.68 |
| Container Index (continuous) |  | 0.93 | 0.88 | 0.99 |  | 0.98 | 0.93 | 1.03 |
| Container Index (categorical) |  | 0.43 | 0.26 | 0.71 |  | 1.02 | 0.66 | 1.58 |
| Potential Container Index (continuous) |  | 1.00 | 0.83 | 1.20 |  | 0.98 | 0.93 | 1.03 |
| Potential Container Index (categorical) |  | 0.90 | 0.55 | 1.48 |  | 0.93 | 0.57 | 1.52 |
| *Stegomyia* Index (continuous) |  | 0.73 | 0.06 | 8.20 |  | 1.00 | 0.99 | 1.00 |
| *Stegomyia* Index (categorical) |  | 0.61 | 0.39 | 0.97 |  | 1.15 | 0.61 | 2.14 |
| * RRs did not converge for these indicators. | | |  |  |  |  |  |  |
