# Supplementary material for: The relationship between entomological indicators of Aedes aegypti abundance and dengue virus infection
Source: PLoS Negl Trop Dis. 2017 Mar 23;11(3):e0005429. doi: 10.1371/journal.pntd.0005429 (PMC5363802; doi:10.1371/journal.pntd.0005429)
Supplement: S6 Table — Adjusted risk ratios (RR) and 95% confidence intervals (CI) for cross-sectional adult stage measures of Ae. aegypti comparing data collected during periods in which the aspirator was changed in 2009: 1999–2008, the CDC backpack aspirator and 2010, the Prokopack aspirator. (DOCX) [file pntd.0005429.s013.docx]

|  |  | **1999-2008 (CDC)** | | |  | **2010 (Prokopack)** | | |
| --- | --- | --- | --- | --- | --- | --- | --- | --- |
| **Indicator** |  | **Risk Ratio** | **95% CI** | |  | **Risk Ratio** | **95% CI** | |
| *Household level* |  |  |  |  |  |  |  |  |
| Adult *Ae. aegypti* (continuous) |  | 1.00 | 0.97 | 1.02 |  | 0.99 | 0.97 | 1.01 |
| Any adult *Ae. aegypti* (categorical) |  | 0.98 | 0.84 | 1.16 |  | 0.79 | 0.63 | 0.99 |
| Adult female *Ae. aegypti* (continuous) |  | 0.98 | 0.95 | 1.03 |  | 0.99 | 0.95 | 1.03 |
| Any adult female *Ae. aegypti* (categorical) |  | 0.96 | 0.80 | 1.16 |  | 0.96 | 0.75 | 1.22 |
| Any adult *Ae. aegypti* indoors (categorical) |  | 1.01 | 0.86 | 1.19 |  | 0.81 | 0.64 | 1.01 |
| Any adult female *Ae. aegypti* indoors (categorical) |  | 0.99 | 0.82 | 1.20 |  | 0.95 | 0.75 | 1.22 |
|  |  |  |  |  |  |  |  |  |
| *Block level* |  |  |  |  |  |  |  |  |
| Adult Premise Index (continuous) |  | 0.99 | 0.98 | 0.99 |  | 1.01 | 1.00 | 1.02 |
| Adult Premise Index (categorical) |  | 0.76 | 0.66 | 0.88 |  | 1.26 | 0.77 | 2.05 |
| Adult Density Index (continuous) |  | 0.79 | 0.62 | 1.01 |  | 1.05 | 0.85 | 1.30 |
| Adult Density Index (categorical) |  | 0.74 | 0.63 | 0.86 |  | 1.40 | 0.49 | 4.01 |
